# Evaluation of the Diagnostic Power of Thermography in Breast Cancer Using Bayesian Network Classifiers

**DOI:** 10.1155/2013/264246

**Published:** 2013-05-22

**Authors:** Cruz-Ramírez Nicandro, Mezura-Montes Efrén, Ameca-Alducin María Yaneli, Martín-Del-Campo-Mena Enrique, Acosta-Mesa Héctor Gabriel, Pérez-Castro Nancy, Guerra-Hernández Alejandro, Hoyos-Rivera Guillermo de Jesús, Barrientos-Martínez Rocío Erandi

**Affiliations:** ^1^Departamento de Inteligencia Artificial, Universidad Veracruzana, Sebastián Camacho 5, Centro, 91000 Xalapa, VZ, Mexico; ^2^Centro Estatal de Cancerología: Miguel Dorantes Mesa, Aguascalientes 100, Progreso Macuiltepetl, 91130 Xalapa, VZ, Mexico; ^3^Laboratorio Nacional de Informática Avanzada (LANIA) A.C. Rébsamen 80, Centro, 91000 Xalapa, VZ, Mexico

## Abstract

Breast cancer is one of the leading causes of death among women worldwide. There are a number of techniques used for diagnosing this disease: mammography, ultrasound, and biopsy, among others. Each of these has well-known advantages and disadvantages. A relatively new method, based on the temperature a tumor may produce, has recently been explored: thermography. In this paper, we will evaluate the diagnostic power of thermography in breast cancer using Bayesian network classifiers. We will show how the information provided by the thermal image can be used in order to characterize patients suspected of having cancer. Our main contribution is the proposal of a score, based on the aforementioned information, that could help distinguish sick patients from healthy ones. Our main results suggest the potential of this technique in such a goal but also show its main limitations that have to be overcome to consider it as an effective diagnosis complementary tool.

## 1. Introduction

 Breast cancer is one of the main causes of death among women worldwide [1]. Moreover, a specificity is required in the diagnosis of such a disease given that an incorrect classification of a sample as a false positive may lead to the surgical removal of the breast [2]. Nowadays, there are different techniques for carrying out the diagnosis: mammography, ultrasound, MRI, biopsies, and, more recently, thermography [3–6]. In fact, thermography started in 1956 [7] but was discarded some years later because of the poor quality of the thermal images [8] and the low specificity values it achieved. However, with the development of new thermal imaging technology, thermography has reappeared and is being seriously considered as a complementary tool for the diagnosis of breast cancer [9]. Because of specificity required, it is compulsory to have as many available tools as possible to reduce, on the one hand, the number of false positives and, on the other hand, to achieve high sensitivity. Although open biopsy is regarded as the gold standard technique for diagnosing breast cancer, it is practically the last diagnostic resource used since it is an invasive procedure that represents not only significant health implications but also psychological and economic ones also [10]. Other techniques, which are not necessarily invasive, have implicit risks or limitations such as X-ray exposure, interobserver interpretability and difficult access to high-tech expensive equipment [11, 12]. Thermography is also noninvasive, but it has the advantage of using a cheaper device (an infrared camera), which is far more portable than those used in mammography, MRI, and ultrasound. Furthermore, it can be argued that some of the variables considered by thermography may be more easily interpreted than those of some of the aforementioned techniques. As a matter of fact, in this paper we will explore and assess this argument in order to measure the potential of such a technique as a diagnostic tool for breast cancer. Moreover, our main contribution is the proposal of a score, based not only on thermographic variables but also on variables that portray more information than temperature alone, that might help differentiate sick patients from healthy ones. We will also explore the potential of thermography in diagnosing women below the age of 50, which would allow the detection of the disease in its early stages, thus reducing the percentage of mortality.

The rest of the paper is divided as follows. In Section 2, we will present some related research that places our research in context and thus appreciates our contribution. In Section 3, we explain the materials and methods used in our experiments. In Section 4, we will present the methodology and the experimental results. In Section 5, we will discuss these results and, finally, in Section 6, we will conclude our paper and give directions regarding future research. 

## 2. Related Research

 In our review of the related literature, we divided these into three categories: introductory, image-based, and data-based works [13–17]. The introductory research mainly points out the potential of thermography as an alternative diagnostic tool for breast cancer comparing its performance to other diagnostic methods such as mammography and biopsy [18, 19]. Unfortunately, because this research is intended as an introduction to the topic, it lacks some important details about the data used in these studies as well as the analyses carried out.

The image-based works mainly range from cluster analyses applied to thermal images (to differentiate healthy from sick breasts) [20] to fractal analyses (to characterize the geometry of the malignant lesions) [21] to the camera calibration for capturing thermal images [3, 22].

The data-based investigations present statistical analyses of patient databases (healthy and sick) such as nonparametric tests, correlation, and analysis of variance; artificial intelligence analyses such as artificial neural networks and Bayesian analysis; and numerical models such as physical and simulation models (bioheat equations) [8, 9, 23–26]. Only a small number of papers propose a score formed from thermographic data [27, 28] but they only propose a maximum of 5 variables to form such a score. In our research, we propose 14 variables to calculate this score: this is the main contribution of the paper alongside the analysis of the diagnostic power of the proposed variables. In Section 3, we will present those variables in more detail and, in Section 4, we will evaluate how informative these variables are in the diagnosis of breast cancer. To end this section, it is important to mention that although the research in this category is very interesting, in some of them the methodology is not clear. This prevents one from easily reproducing the experiments carried out there. We have done our best to present a clear methodology so that our results can be reproduced. 

## 3. Materials and Methods

### 3.1. The Database

 For our experiments, we used a real-world database which was provided by an oncologist who has specialized in the study of thermography since 2008, consisting of 98 cases: 77 cases are patients with breast cancer (78.57%) and 21 cases are healthy patients (21.43%). All the results (either sick or healthy) were confirmed by an open biopsy, which is considered the gold standard diagnostic method for breast cancer [29]. We include in this study 14 explanatory variables (attributes): 8 of them form our score (proposed by the expert), 6 are obtained from the thermal image, one variable is the score itself, and the final variable is age which was discretized in three categories as this is recommended for the selected algorithms [30–32]. In Table 1, we give details of the name, definitions, and values of each of these variables. The dependent variable (class) is the outcome (cancer or no cancer).

### 3.2. Bayesian Networks

 A Bayesian network (BN) [33, 34] is a graphical model that represents relationships of a probabilistic nature among variables of interest. Such networks consist of a qualitative part (structural model), which provides a visual representation of the interactions amid variables, and a quantitative part (set of local probability distributions), which permits probabilistic inference and numerically measures the impact of a variable or sets of variables on others. Both the qualitative and quantitative parts determine a unique joint probability distribution over the variables in a specific problem [33–35]. In other words, a Bayesian network is a directed acyclic graph consisting of [36]: (a) nodes (circles), which represent random variables; arcs (arrows), which represent probabilistic relationships among these variables and (b) for each node, there is a local probability distribution attached to it, which depends on the state of its parents.

Figures 3 and 4 (see Section 4) show examples of a BN. One of the great advantages of this model is that it allows the representation of a joint probability distribution in a compact and economical way by making extensive use of conditional independence, as shown in (1):

(1) $$\begin{array}{c} P\left(X_{1},X_{2},\ldots ,X_{n}\right)=\prod_{i=1}^{n}P\left(X_{i}\;|\;Pa\left(X_{i}\right)\right), \end{array}$$

where *Pa*(*X*_*i*_) represents the set of parent nodes of *X*_*i*_, that is, nodes with arcs pointing to *X*_*i*_. Equation (1) also shows how to recover a joint probability from a product of local conditional probability distributions.

#### 3.2.1. Bayesian Network Classifiers

 Classification refers to the task of assigning class labels to unlabeled instances. In such a task, given a set of unlabeled cases on the one hand and a set of labels on the other, the problem to solve lies in finding a function that suitably matches each unlabeled instance to its corresponding label (class). As can be inferred, the central research interest in this specific area is the design of automatic classifiers that can estimate this function from data (in our case, we are using Bayesian networks). This kind of learning is known as supervised learning [37–39]. For the sake of brevity and the lack of space, we have not written here the code of the 2 procedures used in the tests carried out in this research. We have only briefly described them and refer the reader to their original sources. The procedures used in these tests are (a) the Naïve Bayes classifier, (b) Hill-Climber, and (c) Repeated Hill-Climber [38, 40, 41].

- The Naïve Bayes classifier (NB) is one of the most effective classifiers [38] and the benchmark against which state-of-the-art classifiers have to be compared. Its main appeals lie in its simplicity and accuracy: although its structure is always fixed (the class variable has an arc pointing to every attribute), it has been shown that this classifier has a high classification accuracy and optimal Bayes's error (see Figure 3, Section 4). In simple terms, the NB learns, from a training data sample, the conditional probability of each attribute given the class. Then, once a new case arrives, the NB uses Bayes's rule to compute the conditional probability of the class given the set of attributes selecting the value of the class with the highest posterior probability.
- Hill-Climber is a Weka's [41] implementation of a search and scoring algorithm, which uses greedy-hill-climbing [42] for the search part and different metrics for the scoring part, such as Bayesian information criterion (BIC), Bayesian Dirichlet (BD), Akaike information criterion (AIC), and minimum description length (MDL) [43]. For the experiments reported here, we selected the MDL metric. This procedure takes an empty graph and a database as input and applies different operators for building a Bayesian network: addition, deletion, or reversal of an arc. In every search step, it looks for a structure that minimizes the MDL score. In every step, the MDL is calculated and procedure Hill-Climber keeps the structure with the best (minimum) score. It finishes searching when no new structure improves the MDL score of the previous network.
- Repeated Hill-Climber is a Weka's [41] implementation of a search and scoring algorithm, which uses repeated runs of greedy hill-climbing [42] for the search part and different metrics for the scoring part, such as BIC, BD, AIC, and MDL. For the experiments reported here, we selected the MDL metric. In contrast to the simple Hill-Climber algorithm, Repeated Hill-Climber takes as input a randomly generated graph. It also takes a database and applies different operators (addition, deletion, or reversal of an arc) and returns the best structure of the repeated runs of the Hill-Climber procedure. With this repetition of runs, it is possible to reduce the problem of getting stuck in a local minimum [35].

### 3.3. Evaluation Method: Stratified k-Fold Crossvalidation

 We followed the definition of the crossvalidation method given by Kohavi [37]. In k-fold crossvalidation, we split the database *D* in *k* mutually exclusive random samples called the folds: *D*_1_, *D*_2_,…, *D*_*k*_, where said folds have approximately the same size. We trained this classifier each time *i* ∈ 1,2,…, *k* using *D*∖*D*_*i*_ and testing it on *D*_*i*_ (again, the symbol denotes set difference). The crossvalidation accuracy estimation is the total number of correct classifications divided by the sample size (total number of instances in *D*). Thus, the k-fold crossvalidation estimate is as follows:

(2) $$\begin{array}{c} \text{ac}{\text{c}}_{\text{cv}}=\frac{1}{n}\sum_{\left(v_{i},y_{i}\right)\in D}\delta \left(I\left(D\setminus D_{\left(i\right)},v_{i}\right),y_{i}\right), \end{array}$$

where (*I*(*D*∖*D*_(*i*)_, *v*_*i*_), *y*_*i*_) denotes the label assigned by inducer *I* to an unlabeled instance *v*_*i*_ on dataset *D*∖*D*_(*i*)_, *y*_*i*_ is the class of instance *v*_*i*_, *n* is the size of the complete dataset, and *δ*(*i*, *j*) is a function where *δ*(*i*, *j*) = 1 if *i* = *j* and 0 if *i* ≠ *j*. In other words, if the label assigned by the inducer to the unlabeled instance *v*_*i*_ coincides with class *y*_*i*_, then the result is 1; otherwise, the result is 0; that is, we consider a 0/1 loss function in our calculations of (2). It is important to mention that in stratified k-fold crossvalidation, the folds contain approximately the same proportion of classes as in the complete dataset *D*. A special case of crossvalidation occurs when *k* = *n* (where *n* represents the sample size). This case is known as leave-one-out crossvalidation [37, 39].

For both evaluation methods, we assessed the performance of the classifiers presented in Section 3.2 using the following measures [44–47].

(a) Accuracy: the overall number of correct classifications divided by the size of the corresponding test set:
  (3) $$\begin{array}{c} a=\frac{\text{cc}}{n}, \end{array}$$
  where cc represents the number of cases correctly classified and *n* is the total number of cases in the test set.
(b) Sensitivity: the ability to correctly identify those patients who actually have the disease:
  (4) $$\begin{array}{c} S=\frac{\text{TP}}{\text{TP}+\text{FN}}, \end{array}$$
  where TP represents true positive cases and FN is false negative cases.
(c) Specificity: the ability to correctly identify those patients who do not have the disease:
  (5) $$\begin{array}{c} Sp=\frac{\text{TN}}{\text{TN}+\text{FP}}, \end{array}$$
  where TN represents true negative cases and FP is false positive cases.

## 4. Methodology and Experimental Results

 We used stratified 10-fold crossvalidation on the 98-case database described in Section 3.1. All the algorithms described in Section 3.2.1 used this data in order to learn a classification model. Once we have this model, we then evaluate its performance in terms of accuracy, sensitivity, and specificity. We used Weka [41] for the tests carried out here (see their parameter set in Table 2). For comparison purposes other classifiers were included: a multilayer perceptron (MLP) neural network and decision trees (ID3 and C4.5) with default parameters. The fundamental goal of this experiment was to assess the diagnostic power of the thermographic variables that form the score and the interactions among these variables. To illustrate how the variable values are obtained, we cite one example.

**Figure 1:**
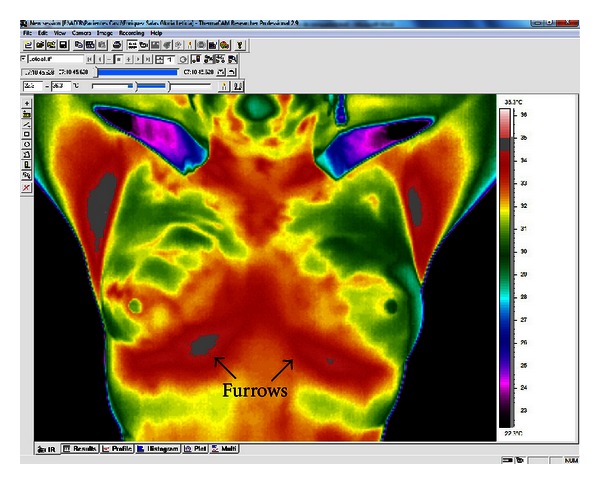
Thermal image showing the temperature of the color-coded breasts. The red and gray tones represent hotter areas.

- In Figure 1 we show the type of images obtained by the thermal imager; in this case, the front of the breast thermography. Using *ThermaCAM Researcher Professional 2.9* [48] software, we detect the hottest areas of the breast that pass from red to gray. The breast whose furrow displays the largest gray area is assigned a positive value and the other a negative one.

**Figure 2:**
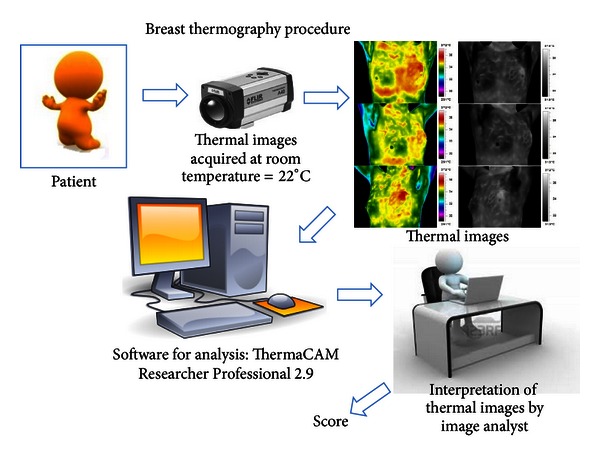
Breast thermography procedure.

In Figure 2 we show a general overview of the procedure of breast thermography, from thermal image acquisition to the formation of the score.

**Figure 3:**
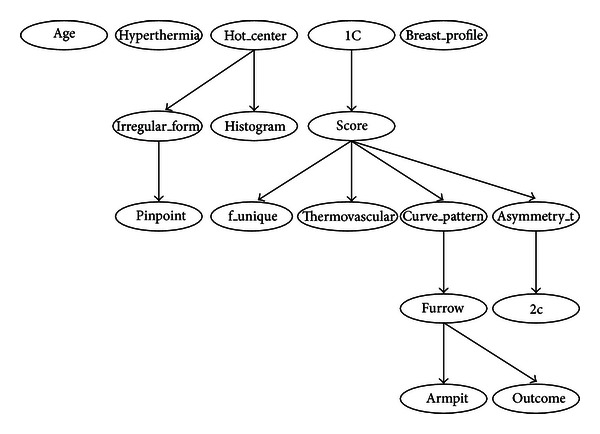
Bayesian network built by procedure of Hill-Climber using the 98-case database. Only variable furrow is directly related to the outcome. Once the variable furrow is known, all the other variables are independent of the class.

**Figure 4:**
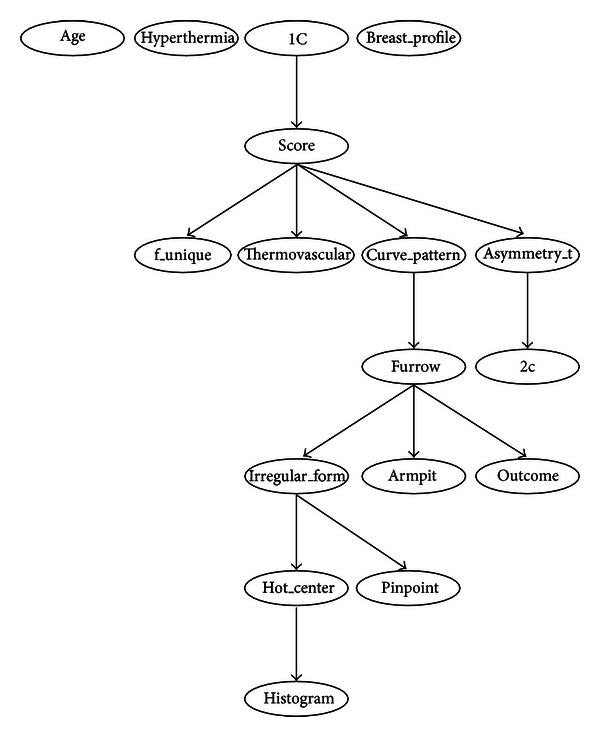
Bayesian network built by procedure of Repeated Hill-Climber using the 98-case database. Only variable furrow is directly related to the outcome. Once the variable furrow is known, all the other variables are independent of the class.

**Figure 5:**
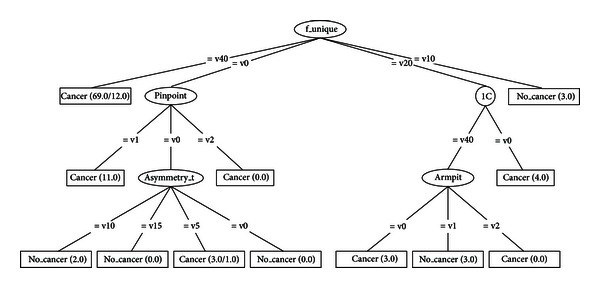
Decision tree C4.5 using the 98-case database.

 Tables 3, 4, 5, 6, 7, 8, 9, and 10 show the numerical results of this experiment. Figures 3 and 4 show the structures resulting from running Hill-Climber and Repeated Hill-Climber classifiers and Figure 5 shows the decision tree (C4.5). We do not present the structure of the Naïve Bayes classifier since it is always fixed: there is an arc pointing to every attribute from the class. For the accuracy test, the standard deviation is shown next to the accuracy result. For the remaining tests, their respective 95% confidence intervals (CI) are shown in parentheses.

## 5. Discussion

 The main objective of this paper is to assess the diagnostic power of thermography in breast cancer using Bayesian network classifiers. As can be seen from Table 3, the overall accuracy is still far from a desirable value. We chose Bayesian networks for the analyses because this model does not only carry out a classification task but it is also able to show interactions between the attributes and the class as well as interactions among the attributes themselves. This ability of Bayesian networks allows us to visually identify which attributes have a direct influence over the outcome and how they are related to one another. The MLP shows a comparable performance but lacks the power of explanation: it is not possible to query this network to know how it reached a specific decision. On the other hand, decision trees do have this explanation capability but lack the power to represent interactions among attributes (explanatory variables). Figures 3 and 4 depict that only 5 variables (out of 16) are directly related to the score: 1C, f_unique, thermovascular, curve_pattern, and asymmetry_t. Hence we can see that the score influence on the class *outcome* is null and the variable *furrow* (this variable is part of the score) is the only one that affects the class. Figure 5 shows that procedure C4.5 also identifies 2 of those 5 variables as being the most informative ones for making a decision: f_unique and asymmetry_t. In fact, if we only consider these attributes, we get the same classification performance as that when taking into account all thermographic variables. Other models, such as artificial neural networks, cannot easily identify this situation. As seen in Section 3.2, the extensive use of conditional independence allows Bayesian networks to potentially disregard spurious causes and to easily identify direct influences from indirect ones. In other words, once these variables are known, they render the rest of the variables independent from the outcome. Another surprising result is that of variable age: some other tests consider this to be an important observation for the diagnosis of breast cancer [30–32]. However, our analyses suggest that, at least with the database used in our experiments, age is not important in a diagnosis when using thermography. As can be seen from Figures 3 and 4, age is disconnected from the rest of the variables. This may imply that thermography shows potential for diagnosing breast cancer in women younger than 50 years of age.

Regarding the sensitivity performance of our models (see Table 3), Hill-Climber and Repeated Hill-Climber achieve a perfect value of 100%. This means that, at least with our database, thermography is excellent for identifying sick patients. Naïve Bayes classifier shows a significantly worse performance; it can be argued that this performance is due to the noise that the rest of the variables may add. Once again, if we only considered the 5 variables mentioned above, we would get the same results as those using Hill-Climber and Repeated Hill-Climber. Other models would not be capable of revealing this situation. Of course, it is mandatory to get more data in order to confirm such results.

It is important to point out that the Hill-Climber and Repeated Hill-Climber procedures identify the same 5 variables as directly influencing the outcome.

Regarding the specificity performance of our models (see Table 3), Hill-Climber and Repeated Hill-Climber achieve the worst possible value of 0%. This means these 5 variables, while being informative when detecting the presence of the disease, are not useful for detecting the absence of such disease (see Tables 5–10). On the other hand, the noise that the rest of the attributes produce when detecting the disease seems to work the other way around: it is not noise but information that makes Naïve Bayes achieve a specificity of 33%. Of course, such a value is far from desirable, but this result makes us think of proposing two different scores (one for sensitivity and one for specificity) with two different sets of variables. But our proposal of a score is a first approximation to combine thermographic variables in such a way as to allow us to tell sick patients from healthy ones. Our results show that such a score needs to be refined in order to more easily identify these types of patients.

Although the results may be discouraging, we strongly believe that they are a step forward in order to more deeply comprehend the phenomenon under investigation: breast cancer. In fact, we have proposed a score that takes into account more information than just that of temperature. Until now, few areas of research have considered other variables apart from that of temperature [27, 28]. Those papers include in their analyses a total of 5 variables that can be extracted from the information a thermogram provides. Our score includes 16 variables and our work, to the best of our knowledge, presents the first analysis of this kind of data using Bayesian networks. What this analysis suggests is a refinement of the score, probably in the sense of proposing a more complex function to represent it beyond the simple addition of the values of each attribute. Intuitively, we thought that other variables, such as hyperthermia or thermovascular network, would be more significant in differentiating sick patients from healthy ones.

In the case of the database, we are aware of the limitations regarding the number of cases and the imbalance of the number of classes. Thus, we would need to collect more data so that more exhaustive tests can be carried out.

## 6. Conclusions and Future Work

 Thermography has been used as an alternative method for the diagnosis of breast cancer since 2005. The basic principle is that lesions in the breasts are hotter than healthy regions. In our experience, only taking into account temperature is not enough to diagnose breast cancer. That is why we proposed a score that considers more information than only temperature alone. We have found that only 5 attributes that are part of this score are the unique direct influence needed to determine if a patient has cancer.

Although some other research projects show better performance than ours, their methodology to carry out the experiments is not clear; thus these experiments cannot be reproduced. Therefore, we need to more closely explore the details of these models and the nature of their data. In this paper we have done our best to present the methodology used in our experiments as clear as possible so that they indeed can be reproduced. It is true that we do not give details about how the database was formed (since this is not the primary goal of the paper). However, we believe that if we make this database available, researchers who want to reproduce our experiments should be able to do so without much trouble.

We have found that the framework of Bayesian networks provides a good model for analyzing this kind of data: it can visually show the interactions between attributes and outcome as well as the interactions among attributes and numerically measure the impact of each attribute on the class.

Although we obtained excellent sensitivity results, we also obtained very poor specificity results. The sensitivity values are consistent with the expectations of the expert, and a discussion about the helpfulness of the Bayesian network is already underway in order to better understand the disease. Given that breast cancer has a special requirement of specificity values, we have to more deeply investigate the causes of those poor results. One possible direction for future research is to collect more balanced data using techniques such as SMOTE [49], ADASYN [50], AdaC1 [51], and GSVM-RU [52]. Another possible direction is to design a more complex score that includes a more complex function compared to that of a simple sum. A third direction we can detect is reviewing how the variables are collected and try to reduce subjectivity in them. Finally, we have also detected that medical doctors usually take into account more information than that supplied to the models for diagnosing breast cancer. Thus, we can also work more in the area of knowledge elicitation.

## Figures and Tables

**Table 1:** Names, definitions, and values of variables. In the experiments the positive value is discretized to 1 and the negative value is discretized to 0. All the values of qualitative variables are given by the image analyst.

Variable name	Definition	Variable value	Variable type
Asymmetry	Temperature difference (in Celsius) between the right and the left breasts	If difference < 1°C, then value = 5, difference between 1°C and 2°C, the value is 10, and difference > 2°C, the value is 15	Nominal (5, 10, 15)

Thermovascular network	Number of veins with the highest temperature	If the visualization is abundant vascularity, the value is 15, if it is moderate, the value is 10, and if it is slight, the value is 5	Nominal (5, 10, 15)

Curve pattern	Heat area under the breast	If heat visualized is abundant, the value is 15, if it is moderate, the value is 10, and if it is slight, the value is 5	Nominal (5, 10, 15)

Hyperthermia	Hottest point of the breast	If there is at least one hottest point, the value is 20 and otherwise the value is 0	Binary (0, 20)

2c	Temperature difference between the hottest points of the two breasts	If difference between 1 and 10, the value is 10, difference between 11 and 15, the value is 15, difference between 16 and 20, the value is 20 and if difference > 20, the value is 25	Nominal (10, 15, 20, 25)

F unique	Amount of hottest points	If sum = 1, the value is 40, if sum = 2, the value is 20, if sum = 3, the value is 10, and if sum > 3, the value is 5	Nominal (5, 10, 20, 40)

1c	Hottest point in only one breast	If the hottest point is only one breast, the value is 40 and if the hottest point is both breasts, the value is 20	Binary (20, 40)

Furrow	Furrows under the breasts	If the furrow is visualized, the value is positive; if not,the value is negative	Binary (0, 1)

Pinpoint	Veins going to the hottest points of the breasts	If the veins are visualized, the value is positive; if not, the value is negative	Binary (0, 1)

Hot center	The center of the hottest area	If the center of the hottest is visualized, the value is positive; if not, the value is negative	Binary (0, 1)

Irregular form	Geometry of the hot center	If the hot center is visualized like a nongeometrical figure, the value is positive; if not, the value is negative	Binary (0, 1)

Histogram	Histogram in form of a isosceles triangle	If the histogram is visualized as a triangle form, the value is positive; if not, the value is negative	Binary (0, 1)

Armpit	Difference temperature between the 2 armpits	If the difference = 0, the value in both is negative; if not, the value is positive; consequently the other is negative	Binary (0, 1)

Breast profile	Visually altered profile	If an altered profile is visualized abundantly, the value is 3, if it is moderate, value is 2, if it is small, the value is 1, and if it does not exist, the value is 0	Binary (0, 1)

Score	The sum of values of the previous 14 variables	If the sum < 160, then the value is negative for cancer; if the sum ≥ 160, the value is positive for cancer	Binary (0, 1)

Age	Age of patient	If the age < 51, the value is 1, if the age between 51 and 71, the value is 2, and if age > 71, the value is 3	Binary (0, 1)

Outcome	The result is obtained via open biopsy	The values are cancer or no-cancer	Binary (0, 1)

**Table 2:** Parameter values for Hill-Climber and Repeated Hill-Climber.

Parameters	Hill-Climber	Repeated Hill-Climber
The initial structure NB (Naïve Bayes)	False	False
Number of parents	100,000	100,000
Runs	—	10
Score type	MDL	MDL
Seed	—	1
Arc reversal	True	True

**Table 3:** Accuracy, sensitivity, and specificity results for the three Bayesian network classifiers presented in Section 3.2.1.

	Naïve Bayes	Hill-Climber	Repeated Hill-Climber
Accuracy	71.88% (±12.61)	76.10% (±7.10)	76.12% (±7.19)
Sensitivity	82% (74–91)	97% (94–100)	99% (96–100)
Specificity	37% (15–59)	0% (0-0)	0% (0-0)

**Table 4:** Accuracy, sensitivity, and specificity of artificial neural network, decision trees ID3 and C4.5 for the thermography.

	Artificial neural network	Decision tree ID3	Decision tree C4.5
Accuracy	67.47% (±15.65)	73.19% (±12.84)	75.50% (±6.99)
Sensitivity	82% (73–91)	87% (79–94)	94% (88–99)
Specificity	33% (13–53)	29% (9–48)	0% (0-0)

**Table 5:** Confusion matrix of Naïve Bayes.

	Cancer	Noncancer	Total
Cancer	TP 65	FN 12	77
Noncancer	FP 14	TN 7	21

			98

TP: true positive, FP: false positive, FN: false negative, TN: true negative.

**Table 6:** Confusion matrix of Hill-Climber.

	Cancer	Non-cancer	Total
Cancer	TP 75	FN 2	77
Non-cancer	FP 21	TN 0	21

			98

TP: true positive, FP: false positive, FN: false negative, TN: true negative.

**Table 7:** Confusion matrix of Repeated Hill-Climber.

	Cancer	Non-cancer	Total
Cancer	TP 76	FN 1	77
Non-cancer	FP 21	TN 0	21

			98

TP: true positive, FP: false positive, FN: false negative, TN: true negative.

**Table 8:** Confusion matrix of artificial neural network.

	Cancer	Non-cancer	Total
Cancer	TP 58	FN 19	77
Non-cancer	FP 15	TN 6	21

			98

TP: true positive, FP: false positive, FN: false negative, TN: true negative.

**Table 9:** Confusion matrix of decision tree ID3.

	Cancer	Non-cancer	Total
Cancer	TP 67	FN 10	77
Non-cancer	FP 15	TN 6	21

			98

TP: true positive, FP: false positive, FN: false negative, TN: true negative.

**Table 10:** Confusion matrix of decision tree C4.5.

	Cancer	Non-cancer	Total
Cancer	TP 76	FN 1	77
Non-cancer	FP 21	TN 0	21

			98

TP: true positive, FP: false positive, FN: false negative, TN: true negative.

## References

[1] Jemal A, Bray F, Center MM, Ferlay J, Ward E, Forman D (2011). Global cancer statistics. *A Cancer Journal for Clinicians*.

[2] Gnerlich J, Jeffe DB, Deshpande AD, Beers C, Zander C, Margenthaler JA (2007). Surgical removal of the primary tumor increases overall survival in patients with metastatic breast cancer: analysis of the 1988–2003 SEER data. *Annals of Surgical Oncology*.

[3] Ng EYK (2009). A review of thermography as promising non-invasive detection modality for breast tumor. *International Journal of Thermal Sciences*.

[4] Bonnema J, Van Geel AN, Van Ooijen B (1997). Ultrasound-guided aspiration biopsy for detection of nonpalpable axillary node metastases in breast cancer patients: new diagnostic method. *World Journal of Surgery*.

[5] Schnall MD, Blume J, Bluemke DA (2005). MRI detection of distinct incidental cancer in women with primary breast cancer studied in IBMC 6883. *Journal of Surgical Oncology*.

[6] Geller BM, Kerlikowske K, Carney PA (2003). Mammography surveillance following breast cancer. *Breast Cancer Research and Treatment*.

[7] Foster KR (1998). Thermographic detection of breast cancer. *IEEE Engineering in Medicine and Biology Magazine*.

[8] Wishart GC, Campisi M, Boswell M (2010). The accuracy of digital infrared imaging for breast cancer detection in women undergoing breast biopsy. *European Journal of Surgical Oncology*.

[9] Arora N, Martins D, Ruggerio D (2008). Effectiveness of a noninvasive digital infrared thermal imaging system in the detection of breast cancer. *American Journal of Surgery*.

[10] Verkooijen HM, Peeters PHM, Buskens E (2000). Diagnostic accuracy of large core needle biopsy for nonpalpable breast disease: a meta-analysis. *British Journal of Cancer*.

[11] Kuhl CK, Schrading S, Leutner CC (2005). Mammography, breast ultrasound, and magnetic resonance imaging for surveillance of women at high familial risk for breast cancer. *Journal of Clinical Oncology*.

[12] Kriege M, Brekelmans CTM, Boetes C (2004). Efficacy of MRI and mammography for breast-cancer screening in women with a familial or genetic predisposition. *New England Journal of Medicine*.

[13] Gutierrez F, Vazquez J, Venegas L Feasibility of thermal infrared imaging screening for breast cancer in rural communities of southern mexico: the experience of the centro de estudios y prevencion del cancer (ceprec).

[14] Hairong Q, Phani TK, Zhongqi L Early detection of breast cancer using thermal texture maps.

[15] Ohsumi S, Takashima S, Aogi K, Usuki H (2002). Prognostic value of thermographical findings in patients with primary breast cancer. *Breast Cancer Research and Treatment*.

[16] Ng EYK, Chen Y, Ung LN (2001). Computerized breast thermography: study of image segmentation and temperature cyclic variations. *Journal of Medical Engineering and Technology*.

[17] Ng EYK, Sudharsan NM (2000). Parametric optimization for tumour identification: bioheat equation using ANOVA and the Taguchi method. *Journal of Engineering in Medicine*.

[18] Sterns EE, Zee B (1991). Thermography as a predictor of prognosis in cancer of the breast. *Cancer*.

[19] Ng EYK, Ung LN, Ng FC, Sim LSJ (2001). Statistical analysis of healthy and malignant breast thermography. *Journal of Medical Engineering and Technology*.

[20] EtehadTavakol M, Sadri S, Ng EYK (2010). Application of K- and fuzzy c-means for color segmentation of thermal infrared breast images. *Journal of Medical Systems*.

[21] EtehadTavakol M, Lucas C, Sadri S, Ng EYK (2010). Analysis of breast thermography using fractal dimension to establish possible difference between malignant and benign patterns. *Journal of Healthcare Engineering*.

[22] Damnjanović Z, Petrović D, Pantović R, Smiljanić Z (2010). Infra red digital imaging in medicine. *International Journal of Collaborative Research on Internal Medicine and Public Health*.

[23] Ng EYK, Fok SC (2003). A framework for early discovery of breast tumor using thermography with artificial neural network. *Breast Journal*.

[24] Ng EYK, Sudharsan NM (2001). Effect of blood flow, tumour and cold stress in a female breast: a novel time-accurate computer simulation. *Journal of Engineering in Medicine*.

[25] Ng EYK, Sudharsan NM (2001). Numerical computation as a tool to aid thermographic interpretation. *Journal of Medical Engineering and Technology*.

[26] Ng EYK, Fok SC, Peh YC, Ng FC, Sim LSJ (2002). Computerized detection of breast cancer with artificial intelligence and thermograms. *Journal of Medical Engineering and Technology*.

[27] Wang J, Chang KJ, Chen CY (2010). Evaluation of the diagnostic performance of infrared imaging of the breast: a preliminary study. *BioMedical Engineering*.

[28] Wang J, Shih TTF, Yen RF (2011). The Association of Infrared Imaging Findings of the Breast with Hormone Receptor and Human Epidermal Growth Factor Receptor 2 Status of Breast Cancer. *Academic Radiology*.

[29] Sardanelli F, Giuseppetti GM, Panizza P (2004). Sensitivity of MRI versus mammography for detecting foci of multifocal, multicentric breast cancer in fatty and dense breasts using the whole-breast pathologic examination as a gold standard. *American Journal of Roentgenology*.

[30] Cross SS, Stephenson TJ, Mohammed T, Harrison RF (2000). Validation of a decision support system for the cytodiagnosis of fine needle aspirates of the breast using a prospectively collected dataset from multiple observers in a working clinical environment. *Cytopathology*.

[31] Cross SS, Dubé AK, Johnson JS (1998). Evaluation of a statistically derived decision tree for the cytodiagnosis of fine needle aspirates of the breast (FNAB). *Cytopathology*.

[32] Walker AJ, Cross SS, Harrison RF (1999). Visualisation of biomedical datasets by use of growing cell structure networks: a novel diagnostic classification technique. *The Lancet*.

[33] Pearl J (1988). *Probabilistic Reasoning in Intelligent Systems: Networks of Plausible Inference*.

[34] Neuberg LG (2003). Causality: models, reasoning, and inference, by judea pearl, cambridge university press, 2000. *Econometric Theory*.

[35] Friedman N, Goldszmidt M (1998). *Learning Bayesian Networks from Data*.

[36] Cooper G (1999). An overview of the representation and discovery of causal relationships using bayesian networks. *Computation Causation Discovery*.

[37] Kohavi R (1995). *A Study of Cross-Validation and Bootstrap for Accuracy Estimation and Model Selection*.

[38] Friedman N, Geiger D, Goldszmidt M (1997). Bayesian Network Classifiers. *Machine Learning*.

[39] Han J, Kamber M (2006). *Data Mining: Concepts and Techniques*.

[40] Duda RO, Hart PE, Stork DG (2001). *Pattern Classification*.

[41] Witten IH, Frank E (2005). *Data Mining: Practical Machine Learning Tools and Techniques*.

[42] Russell SJ, Norvig P (2009). *Artificial Intelligence: A Modern Approach*.

[43] Tsamardinos I, Brown LE, Aliferis CF (2006). The max-min hill-climbing Bayesian network structure learning algorithm. *Machine Learning*.

[44] Lavrač N (1999). Selected techniques for data mining in medicine. *Artificial Intelligence in Medicine*.

[45] Cross SS, Dubé AK, Johnson JS (1998). Evaluation of a statistically derived decision tree for the cytodiagnosis of fine needle aspirates of the breast (FNAB). *Cytopathology*.

[46] Cross SS, Stephenson TJ, Mohammed T, Harrison RF (2000). Validation of a decision support system for the cytodiagnosis of fine needle aspirates of the breast using a prospectively collected dataset from multiple observers in a working clinical environment. *Cytopathology*.

[47] Cross SS, Downs J, Drezet P, Ma Z, Harrison RF (2000). *Which Decision Support Technologies are Appropriate for the Cytodiagnosis of Breast Cancer?*.

[48] Inc FLIR System (2009). Thermacam researcher professional 2.9. http://support.flir.com/DocDownload/Assets/47/English/T5590091387.

[49] Bowyer KW, Chawla NV, Hall LO, Kegelmeyer WP (2002). Smote: synthetic minority over-sampling technique. *Journal of Artificial Intelligence Research*.

[50] He H, Bai Y, Garcia EA, Li S Adasyn: adaptive synthetic sampling approach for imbalanced learning.

[51] Sun Y, Kamel MS, Wong AKC, Wang Y (2007). Cost-sensitive boosting for classification of imbalanced data. *Pattern Recognition*.

[52] Tang Y, Zhang YQ Granular SVM with repetitive undersampling for highly imbalanced protein homology prediction.

